# The Role of Perfectionism and Controlling Conditions in Norwegian Elite Junior Performers’ Motivational Processes

**DOI:** 10.3389/fpsyg.2019.01366

**Published:** 2019-06-12

**Authors:** Heidi Marian Haraldsen, Hallgeir Halvari, Bård Erlend Solstad, Frank E. Abrahamsen, Sanna M. Nordin-Bates

**Affiliations:** ^1^Department of Coaching and Psychology, Norwegian School of Sport Sciences, Oslo, Norway; ^2^Norwegian Research Center of Children and Youth Sports, Norwegian School of Sport Sciences, Oslo, Norway; ^3^Department of Business, Marketing and Law, University of South-Eastern Norway, Kongsberg, Norway; ^4^Department of Performance and Training, Swedish School of Sport and Health Sciences, Stockholm, Sweden

**Keywords:** self-determination theory, motivation, perfectionism, teaching style, controlling conditions, talent development, performance

## Abstract

Conceptualized within the framework of self-determination theory, the aim of the current study was to investigate the relation between perfectionistic concerns and (a) controlled (non-self-determined) motivation and (b) performance anxiety through basic psychological need frustration (frustration of competence, autonomy, and realtedness), and if these relations would be moderated by controlling teaching/coaching conditions. We used a cross-sectional moderated mediation design and purposefully selected Norwegian elite junior performers (*N* = 171; mean age = 17.3; SD age = 0.94) from talent development schools, who completed an online questionnaire to report their perceptions of the study variables. Associations were examined using structural equation modeling. The results showed that perfectionistic concerns were positively associated with controlling conditions, basic needs frustration, controlled motivation, and performance anxiety. Reported controlling teaching/coaching conditions moderated the positive indirect relationship between perfectionistic concerns and (a) controlled motivation and (b) performance anxiety through competence need frustration. Specifically, these indirect associations were evident for performers reporting moderate or high levels of controlling teaching/coaching conditions. In contrast, there were no indirect associations via competence need frustration for those performers who reported low levels of controlling conditions. In conclusion, the results indicate that perfectionistic concerns appear to be a vulnerability factor that exposes elite junior performers to higher risks of entering a debilitative motivational process. This seems especially likely when exposed to controlling teaching/coaching conditions. Coaches and teachers working with elite junior performers should avoid using controlling mechanisms and instead foster autonomous functioning.

## Introduction

Elite junior performers in sport and performing art are at increased risk for poor functioning and ill-being compared to the average population, due to the unique requirements associated with reaching excellence (Hill A. et al., 2016; Mainwaring and Finney, 2017; Drew et al., 2018). This urges scholars to address risk factors (e.g., traits and conditions) to better safeguard talent development environments (TDEs). Perfectionism is such a trait risk factor found to be more common in elite performers (Dunn et al., 2012). Particularly, perfectionistic concerns (PC) are considered a vulnerability factor associated with higher levels of controlled motivation (i.e., extrinsically regulated behavior) and performance anxiety (Stoeber et al., 2007; Hill A.P. et al., 2016; Patston and Osborne, 2016). To understand why and under what circumstances elite junior performers reporting PC are at risk of experiencing controlled motivation and performance anxiety, we applied the conceptual framework of self-determination theory (SDT; Ryan and Deci, 2017). Specifically, we wanted to examine the potential roles of controlling teaching/coaching conditions and basic psychological need frustration (i.e., need for competence, autonomy, and relatedness) as explaining mechanisms.

Perfectionism is a trait defined as the desire to reach very high standards accompanied by overly self-critical evaluations (Frost et al., 1990; Hill, 2016). Perfectionistic concerns (PC), a sub-dimension of perfectionism, are characterized by combinations of concern over mistakes, doubts about actions, and fear of negative social evaluation, regardless of achievements (Hill, 2016). Paradoxically, PC energize a strong motivational force to strive (i.e., focus, persistence, and discipline), yet, the rigid over-striving attitude, directed toward seeking approval, avoiding mistakes, and maintaining self-worth, also facilitates debilitative patterns of cognition, affect, and behavior (DiBartolo et al., 2004; Appleton and Curran, 2016; Patston and Osborne, 2016). Indeed, research evidence concerning PC shows consistent positive associations with a range of maladaptive outcomes, such as controlled motivation, performance anxiety, and achievement challenges (Gotwals et al., 2012; Hill A.P. et al., 2016).

Performance anxiety is defined as experienced stress before and during performance, often because of an apparent imbalance between situational demands and the perceived competence to counter the requests (Lazarus, 2000; Correia and Rosado, 2018). Performance anxiety comprises cognitive anxiety (i.e., negative self-talk, catastrophizing), somatic anxiety (i.e., increased heart rate, muscle tension), and self-confidence (i.e., doubts in one’s abilities; Cox and Russell, 1999). TDEs are likely to be stressful (e.g., high expectations, social evaluation, and deselection), generally nurturing performance anxiety in both elite and elite junior performers (Patston and Osborne, 2016). Furthermore, there is support for a positive relation between PC and performance anxiety, and high levels of PC have been associated with higher risk of developing performance anxiety (Patston and Osborne, 2016). When displaying high levels of PC, the tendency to feel inadequate and self-critical constantly threatens the balance between demands and perceived competence. Moreover, PC seem to affect the cognitive dimension of anxiety most strongly (Miller and Chesky, 2004; Walker and Nordin-Bates, 2010). The accompanying doubt, worry, and negative self-talk that follows PC when facing risk of failure, have been found to activate stress and avoidance coping strategies (Lazarus, 2000; Hill A.P. et al., 2016). Hence, performers with PC seem to lack growth-seeking and proactive behavior when confronted with stress, thereby being even more vulnerable when participating in TDEs (Stoeber and Eismann, 2007; Hill A.P. et al., 2016).

Although the relationship between PC and performance anxiety is well documented, the explanatory mechanisms involved have been understudied (Boone et al., 2014). Given that PC is considered a general vulnerability factor for a broad range of maladaptive outcomes (e.g., controlled motivation, performance anxiety, and burnout: Hill A.P. et al., 2016), focusing on more broad dynamics involved in PC might help extend the perfectionism literature. Hence, this study is building on previous studies applying the general theoretical framework of SDT (Boone et al., 2014; Jowett et al., 2016) and testing some core motivational concepts (controlling conditions and basic psychological needs) as explanations of *why* and *when* debilitative processes occur (Vansteenkiste and Ryan, 2013; Ryan and Deci, 2017).

A central tenet of SDT is that the satisfaction of the three basic psychological needs, nurtures psychological growth and well-being (Ryan and Deci, 2017). Conversely, need frustration underpins a range of malfunctioning and ill-being constructs (Vansteenkiste and Ryan, 2013; Haerens et al., 2015; Ryan and Deci, 2017). These needs are competence (feeling mastery when interacting with one’s environment), autonomy (experiencing volition, and acting in accordance with one’s true self), and relatedness (experiencing a mutual connectedness with others; Vansteenkiste and Ryan, 2013). When experiencing need frustration, the needs would manifest in feelings of inferiority and failure (competence need frustration), pressure and manipulation (autonomy need frustration), and distance and isolation (relatedness need frustration; Haerens et al., 2015).

A recent meta-analysis found that PC were consistently associated with need frustration (Hill and Curran, 2016). Given the ultimate goal of demonstrating outstanding performance, and the competitive nature of TDEs, failure seems at least as likely an outcome as success for elite junior performers. Hence, the need for competence seems to be especially at risk of not being satisfied in TDEs. When displaying PC, one’s competence evaluation is often biased (Shafran et al., 2002); self-critical and harsh when faced with failure, and underestimated and re-evaluated when faced with success. In addition, PC are associated with a lack of reactivity patterns to cope with adversity (Flett and Hewitt, 2016). Hence, frustration of competence might be the outcome, independently of any objectively achieved results. PC are also associated with rigid and controlled behavior regulations (i.e., “must,” “have to,” and “should”), which might be out of line with autonomous and creative functioning (Hall, 2016; Hill, 2016). Lastly, PC are associated with obsessiveness, social comparisons, and interpersonal inflexibility (indicative of frustration of the need for relatedness), underpinned by a narrow-minded and competitive dedication (Boone et al., 2014; Hall, 2016). As such, frustration of the three basic psychological needs seems likely to be nurtured by PC.

The negative consequences of long-term need frustration are evident in prior SDT-based studies, associated with low quality of motivation (e.g., controlled motivation) and various forms of malfunction and ill-being (Vansteenkiste and Ryan, 2013; Haerens et al., 2015; Bartholomew et al., 2018). For example, in a study focusing on resilience processes after experienced need frustration, restoration was nurtured by autonomous functioning and moderated by perceived competence (Radel et al., 2013). In light of the characteristics of PC, a proactive ability to engage in resilience processes and restore the basic needs when frustrated seems to be lacking when experiencing high levels of PC (Vansteenkiste and Ryan, 2013; Hill A.P. et al., 2016).

To date, some evidence of positive associations between PC and need frustration has been found (Hill A.P. et al., 2016). Recent studies have shown that PC, through general need frustration, were indirectly linked to symptoms of burnout (Jowett et al., 2016) and binge eating (Boone et al., 2014). Despite studies having successfully examined relations between the need for competence and motivation, performance, and well-being (Fransen et al., 2018a,b), no studies, to our knowledge, have focused on the indirect links between PC and such outcomes through each need separately. In addition, no study has tested whether such indirect associations are conditional on specific environmental aspects, such as controlling teaching/coaching style.

An important area of inquiry, suggested to extend perfectionism research (Appleton and Curran, 2016), is factors that contribute to explain the development of perfectionism (e.g., the social environment). The pressure of being perfect is proposed to originate from exposure to psychological control (e.g., manipulation through expectations, criticism, and conditional love) imposed by social agents, such as parents (Soenens and Vansteenkiste, 2010). Thus, perfectionistic behaviors seem to compensate for internal feelings of inadequacy, inferiority, and low self-worth by seeking external approval and acceptance (Eusanio et al., 2014; Flett and Hewitt, 2016). The same contingent mechanisms and patterns underlying the child-parent relationship, might be extended and re-visited in adolescence in interpersonal relationships developing in TDEs, such as those with teachers and coaches (Soenens and Vansteenkiste, 2010). Research from sport psychology has found that social agents using psychological control seem particularly important in the development of the PC aspects of perfectionism that are linked to conditional and unstable self-worth (i.e., fear of negative social evaluation and concern of mistakes; Appleton and Curran, 2016; Hill, 2016).

In the SDT-based literature, controlling teaching/coaching style is characterized by the use of conditional regard, meaning that approval and acceptance are given only when students behave or live up to the expected and preconceived standards of emotions, cognitions, and behavior (Reeve, 2009; Assor et al., 2014; Bartholomew et al., 2018). Such controlling teaching/coaching may be represented by humiliation, yelling, critique, or punishment, which have been found to nurture external motivational regulations (Soenens and Vansteenkiste, 2010; De Meyer et al., 2016). The experience of pressure and control might also work indirectly via attention withdrawal or showing disappointment, which in turn, may create guilt, shame, self-criticism, and anxiety (Soenens and Vansteenkiste, 2010; Bartholomew et al., 2018). These experiences are likely to generate introjected motivational regulations that control the way of thinking and acting from inside the person (Soenens and Vansteenkiste, 2010). It is worth noting that both introjected and external motivational regulations are characterized as controlled motivation within the SDT-based literature. They are associated with less engagement and persistence, and with the lack of proactive coping strategies (Mouratidis and Michou, 2011; Ryan and Deci, 2017). Hence, controlled motivation is likely to be negative for elite performance (Soenens et al., 2012). PC performers are likely to experience the teaching/coaching style with a biased mindset (Shafran et al., 2002; Nordin-Bates et al., 2014), monitoring for critical feedback, lack of attention, and other signs of imperfection or disapproval. Hence, performers reporting higher levels of PC might be more susceptible to the development of controlled motivation and associated outcomes (e.g., performance anxiety) in highly controlling teaching/coaching conditions (Haerens et al., 2015; Appleton and Curran, 2016).

Despite the empirical evidence in relation to controlling teaching/coaching behaviors, such a teaching/coaching style still appears to be a common phenomenon in TDEs (Reeve, 2009; De Meyer et al., 2016; Bartholomew et al., 2018). Research has also indicated that controlling conditions are likely to be found within experience-based and top-down apprenticeship cultures (e.g., arts and sports), in which the teachers/coaches are seen as authority figures (i.e., former top performers) and, in some cases, gatekeepers who are holding significant power over their students/athletes (Lakes, 2005; Nash and Collins, 2006; Burwell, 2013). Few studies, however, have investigated the role of controlling conditions within TDE’s including elite junior performers of these performance domains.

**FIGURE 1:**
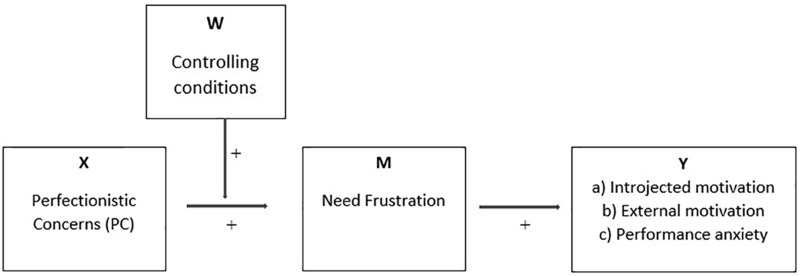
Proposed moderated mediation model.

Based on the research reviewed and SDT-based tenets, the present study tested the following hypotheses (see also Figure 1):

1. PC are positively related to controlling conditions, need frustration, introjected motivation, external motivation, and performance anxiety.
2. Controlling conditions will moderate the relation between PC and frustration of the needs for autonomy, competence, and relatedness, in such a manner that these relationships will be positive and stronger among those who report higher levels of controlling teaching/coaching conditions.
3. The indirect associations between PC and (a) introjected motivation, (b) external motivation, and (c) performance anxiety via the frustration of autonomy, competence, and relatedness will be more evident among those who report higher levels of controlling teaching/coaching conditions.

## Materials and Methods

### Participants and Ethical Considerations

We purposefully recruited and invited all high-achieving elite junior performers (achieving within top 20%) who also attended prestigious junior TDE schools across selected activities in sport and arts in Norway at age 16–19 (*M* = 17.31; *SD* = 0.94). The 171 participants (84 boys, 87 girls) came from individual sports (*N* = 118; swimming, rowing, athletics, skating, cross-country skiing, biathlon, and alpine skiing) and art (*N* = 59; classical music and ballet). The TDEs in sport were operated by the sports federations in collaboration with the Norwegian Olympic Center and specialized private high schools for elite sports, while specialized higher education institutions ran the TDE schools (conservatoires) within the arts. All programs had entrance regulated by competitive auditions, and offered both acceleration and enrichment. The study gained a response rate of 84%, and thus, represent a unique sample of the best junior performers present in the small country of Norway (about 5 million inhabitants). Other studies of successful versus less successful elite performers across domains have found that elite performers are distinctive, sharing many similar psychological characteristics (Ericsson et al., 2003). The performers had all participated in deliberate practice in their activity for many years (*M* = 9.56; *SD* = 3.21). Moreover, they spent many hours on their activity each week (*M* = 20.92; *SD* = 7.98).

We recruited the participants through a dialogue with sport federations, national teams, and leaders of TDE schools. They voluntarily and in writing consented to participate in accordance to the Declaration of Helsinki, after receiving oral and/or written information about the study. This study was carried out after ethical approval of the protocol by the state governed Norwegian Center for Research Data (approval code nr. 53471). The data was collected using a digital survey tool called SurveyXACT, and the participants received a personal link by email. In collaboration with the sport federations and TDE art schools, the first author traveled to collect the data directly in separate activity groups, which helped monitor the data collection settings. For some participants, however, the survey was answered privately due to a lack of scheduled national team practices or due to absence. Finally, the data was transferred to IBM Statistics SPSS 24.0 and M*plus* version 8 for data analyses.

### Measurements

All measurements are based on translated, contextualized, piloted, and validated questionnaires. To contextualize the measurements the first author translated the questionnaires to Norwegian, the fourth author performed a back-translation and both adjusted the final version. The contextualization was executed by instructional information, “tagging” in front of each item section, as well as contextualized adaptation on item-level where it was natural to do so (Madigan and Stoeber, 2016). We then tested a pilot version of the questionnaire on two former TDE performers who gave feedback on the given use of language, contextualization, and instructions, before administering the survey.

#### Perfectionistic Concerns

A contextualized version of the Frost Multidimensional Perfectionism Scale was used (F-MPS; Frost et al., 1990). The subscales Concern over Mistakes (CM, nine items; e.g., “If I fail at my activity, I feel like a failure as a person”) and Doubts about Actions (DA – four items; e.g., “It takes me a long time to do something “right”) assessed perfectionistic concerns. The participants answered on a 7-point Likert scale from 1 (*totally disagree*) to 7 (*totally agree*). This scale has been shown reliable and valid in several studies, including in contextualized versions in sport and art (Madigan and Stoeber, 2016).

#### Controlling Conditions

The Perceived Controlling Style Scale (Halvari et al., 2012), was used (six items; e.g., “I experience that my teacher/coach is making all the decisions”). Responses were made on a 5-point Likert scale from 1 (*totally disagree*) to 5 (*totally agree*). The initial validation study supported the internal consistency and factor structure of the scale (Halvari et al., 2012).

#### Need Frustration

The Basic Psychological Need Satisfaction and Frustration Scale (Chen et al., 2015) was adapted to measure need frustration. Four items captured need frustration for each of competence (e.g., “I feel insecure regarding my ability to master my activity”), autonomy (e.g., “Most of the things I do feel like “I have to”), and relatedness (e.g., “I feel the relationships I have are just superficial”). The subscales were measured on a 7-point Likert scale from 1 (*totally disagree*) to 7 (*totally agree*). This scale has been validated and assessed across contexts and cultures (Chen et al., 2015).

#### Controlled Motivation

The Behavioral Regulations in Sport Questionnaire (BRSQ; Lonsdale et al., 2008) subscales of introjected regulation (four items; e.g., “I would feel ashamed if I quit”) and external regulation (e.g., “I feel pressure from other people to participate in my activity”) was used. The responses were made on a 7-point Likert scale from 1 (*totally disagree*) to 7 (*totally agree*). The instrument has been developed and shown to be valid in sport contexts, as well as in art contexts (Hancox et al., 2015).

#### Performance Anxiety

The Mental Readiness Form (MRF-3; e.g., Krane, 1994) assessed performance anxiety related to competitive situations (i.e., competition or stage performance). This is a short form of only three items, designed and validated (Cox and Russell, 1999) to correspond with subscales of cognitive anxiety, somatic anxiety, and self-confidence from the Competitive State Anxiety Inventory (Martens et al., 1990). Responses were made on a scale ranging from 1 to 100% of anxiety arousal (divided by 10 in the analyses) to assess the participants‘ experienced anxiety levels.

### Analytical Strategy

The data were first checked for normality, missing values, and outliers (Tabachnick and Fidell, 2007). To validate the measures we tested factor loadings and model fit using confirmatory factor analyses (CFA) in M*plus* version 8. If the validation failed, we did supplemental explorative factor analysis (EFA) in SPSS to explore how data adjusted to the expected theoretical subscales in our sample and searched for reduced, but theoretical meaningful subscales. Finally, we calculated reliability values for each scale in M*plus* using coefficient omega, found more appropriate for most research applications (Widaman et al., 2011).

Next, we calculated descriptive statistics and bivariate correlations using SPSS. The Spearman ρ was applied, as dichotomous controlling variables (gender, domain) were included, and as it has been found more robust to a lack of normal distribution (Tabachnick and Fidell, 2007). Cohen’s evaluation of small (*r* = 0.10–0.29), medium (*r* = 0.30–0.49), and large effects (*r* > 0.50) were used for interpretation (Cohen et al., 2003).

For the main analyses, we applied moderated mediation (Hayes, 2017; Muthén et al., 2017). To extend the popular mediation models scholars have suggested that it may be wise to determine if an association is constant across different contexts, groups or characteristics of individuals, or contingent of the interaction with circumstances (Hayes, 2017). We therefore first conducted simple moderation analysis in SPSS using Hayes (2017) model templates with mean-centered product variables. This analysis explored the contribution of the direct and interaction associations of PC and controlling conditions on the intervening variables (each need frustration), and to receive beta coefficients to probe and visualize the interactions. This procedure was repeated in three models for each need separately.

Structural Equation Modeling (SEM) was chosen for the final analyses of the full models as it also provides model fit indices, bootstrap confidence intervals (CI), and strategies for dealing with missing data. For reasons of parsimony and to increase statistical power, we estimated the model containing only one intervening variable and one outcome variable at a time. Aligned with critique raised toward estimation of interaction of latent variables (Hayes, 2017), and as the sample size of the current study may be regarded as low for latent variables modeling (*N* = 171), manifested variables were used in the SEM models to ensure sufficient statistical power (Cohen et al., 2003; Schweizer and Furley, 2016). An *a priori* sample size calculator for multiple regression (Soper, 2018) recommended minimum 97 participants to reach a power level of 0.8 to detect an effect size of 0.15, at an alpha level of 0.05 and with six variables. As suggested by previous research (e.g., Marsh et al., 2004), good model fit is indicated by a chi-square non-significant *p*-value (>0.05). As the chi-square test can be sensitive to sample size, however, the relative chi-square (χ^2^/df <2) is a robust supplemental test (Marsh et al., 2004). For additional fit evaluation, we relied on both incremental (CFI) and absolute (RMSEA/SRMR) indices. Fit was deemed acceptable if RMSEA/SRMR values were close to or lower than 0.08, accompanied by a CFI value close to or higher than 0.95 (Marsh et al., 2004).

## Results

### Preliminary Analyses

#### Screening and Validation

There were no outliers and few missing data (0.6–1.7%). The missing data were handled using Full Information Maximum Likelihood, claimed to be a robust strategy (Lang and Little, 2018). As the variables were moderately skewed (range -0.04 to 1.09) and kurtosis (range -0.04 to 1.16; e.g., Tabachnick and Fidell, 2007), as expected in a high-achieving sample, 10.000 bootstrap was conducted in all analysis as advised by previous researchers (Ng and Lin, 2016).

An overall CFA of all the study variables showed acceptable fit [χ^2^(565) = 860.13, *p* = 0.00, χ^2^/df = 1.5, CFI = 0.90, SRMR = 0.06, RMSEA = 0.06 (90% CI, 0.05–0.06)] after some adjustments in the validation process of each sub-scale. Especially the concern over mistakes sub-scale of PC had to be reduced and adjusted (for details of the instrument validation, see Supplementary Material).

#### Descriptive Statistics and Bivariate Correlations

Table 1 presents means, standard deviations, reliability estimates, and inter-correlations for all study variables, including domain and gender. As shown, the performers tended to display moderate levels of PC, low levels of controlling conditions, basic needs frustration, controlled motivation, and moderate levels of performance anxiety. In line with hypothesis 1, the correlations revealed that PC shared medium to large positive associations with all other variables.

**Table 1:** Descriptive statistics and estimated correlation matrix (Spearman’s rho) for the study variables.

Variable	M (SD)	Ω	1	2	3	4	5	6	7	8	9	10
1. Perfectionistic concerns	3.36 (1.1)	0.82	–									
2. Controlling conditions	1.83 (0.7)	0.75	0.43**	–								
3. Frustration competence	2.37 (1.4)	0.86	0.59**	0.45**	–							
4. Frustration autonomy	2.33 (1.4)	0.87	0.49**	0.44**	0.68**	–						
5. Frustration relatedness	1.90 (1.3)	0.88	0.50**	0.42**	0.58**	0.65**	–					
6. Introjected motivation	3.11 (1.8)	0.86	0.48**	0.38**	0.56**	0.55**	0.63**	–				
7. External motivation	1.96 (1.3)	0.88	0.37**	0.40**	0.51**	0.55**	0.63**	0.75**	–			
8. Performance anxiety	3.74 (2.3)	0.75	0.33**	0.25**	0.36**	0.26**	0.24**	0.30**	0.27**	–		
9. Domain	–	–	–0.24*	–0.14*	–0.29**	–0.25**	–0.25**	–0.15	–0.11	–0.20*	–	
10. Gender	–	–	0.20**	0.05	0.10	0.03	0.12	0.10	0.06	0.19**	–0.12	–


*^∗^p < 0.05, ^∗∗^*p* < 0.01 (2-tailed); M, mean; SD, standard deviation; Ω, omega coefficient. All scales are measured on a 7-point Likert scale except controlling conditions (5-point Likert scale) and performance anxiety (1–100% arousal divided by 10). Domain refers to art (= value 1) vs. sport (= value 2). Gender refers to boys (= value 1) vs. girls (= value 2).*

#### Controlling Variables

Analysis of variance (ANOVA) was performed to examine potential differences between gender and domain (sport vs. art) on the key study variables. The results indicated significant effects by gender on PC (*F* = 6.18, *df* = 1, *p* = 0.01, $\eta_{p}^{2}$ = 0.04), frustration of relatedness, (*F* = 4.62, *df* = 1, *p* = 0.03, $\eta_{p}^{2}$ = 0.03), and anxiety, (*F* = 7.24, *df* = 1, *p* = 0.01, $\eta_{p}^{2}$ = 0.04). Girls reported higher scores than boys for all these variables, with small ($\eta_{p}^{2}$ >0.01 <0.06) effects (Fritz et al., 2012). Domain also showed significant and small to moderate ($\eta_{p}^{2}$>0.06) effects for PC (*F* = 10.10, *df* = 1, *p* = 0.00, $\eta_{p}^{2}$ = 0.06); competence frustration (*F* = 16.34, *df* = 1, *p* = 0.00, $\eta_{p}^{2}$ = 0.09); autonomy frustration (*F* = 8.66, *df* = 1, *p* = 0.00, $\eta_{p}^{2}$ = 0.05); relatedness frustration (*F* = 11.63, *df* = 1, *p* = 0.00, $\eta_{p}^{2}$ = 0.07); and anxiety (*F* = 7.24, *df* = 1, *p* = 0.01, $\eta_{p}^{2}$ = 0.04). Sport performers scored lower on these variables compared to art performers. Due to these results, and to keep the main model as parsimonious as possible, domain was added as a categorical control variable associated with the intervening variables (need frustration), whereas domain and gender were added as categorical control variables associated to the outcomes (introjected, external, and anxiety) to control for their influence on the model results.

### Main Analyses

#### Moderation

**FIGURE 2:**
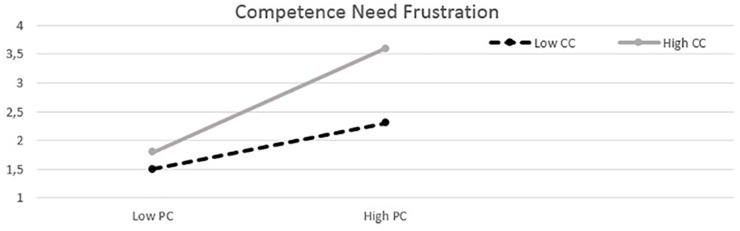
Competence need frustration (*Y*-axis) as a function of reported perfectionistic concerns (PC) and controlling conditions (CC). Low refers to –1 SD below the mean, whereas high refers to +1 SD above the mean.

Hypothesis 2 suggested that controlling conditions would moderate positively the relation between PC and each need frustration, such that this association would be stronger for those who reported higher, instead of lower, levels of controlling conditions. However, the analyses using PC as an independent variable and controlling conditions as a moderator toward each need as dependent variables, showed only support for the moderation model on need for competence (PC/b_1_x = 0.53, *p* = 0.00; Control/b_2_w = 0.55, *p* = 0.00; PC^∗^control/b_3_xw = 0.29, *p* = 0.01; *R*^2^ = 0.45). In the cases of frustration of need for autonomy (PC/b_1_x = 0.38, *p* = 0.00; Control/b_2_w = 0.59, *p* = 0.00, PC^∗^control/b_3_xw = 0.13, *p* = 0.30; *R*^2^ = 0.27) and relatedness (PC/b_1_x = 0.32 *p* = 0.00; Control/b_2_w = 0.52, *p* = 0.00; PC^∗^control/b_3_xw = 0.16, *p* = 0.158; *R*^2^ = 0.28), no significant interactions were present. In summary, these moderation analyses showed that hypothesis 2 was supported only in the model of frustration of competence. Specifically, as visualized in Figure 2, competence frustration was stronger for those experiencing higher levels of controlling conditions, and this difference increased when PC increased (calculated from equation: ỹ = ìy +b1x +b2w’ +b3xw’, with -1 SD below the mean as low, and 1 SD above the mean as high values; Hayes, 2017). The additional *t*-tests with the Johnson-Neyman technique (Hayes, 2017) for the model of competence frustration showed that the range of statistical significance covered the entire variety of the moderator values in the data from the lowest score 1 (*t* = 2.43, *p* = 0.02) to the highest score 4.2 (*t* = 4.22, *p* = 0.00). Due to these results, the competence need frustration (CNF) was decided to be the only intervening variable used while testing hypothesis 3 in the further moderated mediation analyses.

#### Moderated Mediation

Complete moderated mediation results are presented in Table 2. The models provided very good fit indices for the models of introjected motivation^a^, χ^2^(1) = 0.10, *p* = 0.76, χ^2^/df = 0.05, CFI = 1.00, SRMR = 0.03, RMSEA = 0.00 (90% CI, 0.00–0.14), external motivation^b^, χ^2^(1) = 0.10, *p* = 0.74, χ^2^/df = 0.05, CFI = 1.00, SRMR = 0.03, RMSEA = 0.00 (90% CI, 0.00–0.14), and performance anxiety^c^, χ^2^(1) = 0.88, *p* = 0.77, χ^2^/df = 0.19, CFI = 1.00, SRMR = 0.03, RMSEA = 0.00 (90% CI, 0.00–0.14). The effect sizes of explained variance of the intervening variable CNF (*R*^2^ = 0.45), as well as for the outcomes (a) introjected motivation (*R*^2^ = 0.32), (b) external motivation (*R*^2^ = 0.29), and (c) performance anxiety (*R*^2^ = 0.32) were large (Fritz et al., 2012). The results showed direct associations from PC on introjected motivation (β = 0.18, *p* = 0.02) and performance anxiety (β = 0.20, *p* = 0.03), but not on external motivation (β = 0.00, *p* = 0.96). In contrast, direct associations were found from controlling conditions on external motivation (β = 0.23, *p* = 0.003), but not on introjected motivation (β = 0.11, *p* = 0.158) and performance anxiety (β = 0.04, *p* = 0.654). There was no significant direct interaction effects (PC^∗^Controlling conditions) associated with the three outcomes in any of the models. However, the index of the conditional indirect effects between PC and (a) introjected motivation [*index* = 0.29 (95% CI, 0.10–0.57), *p* = 0.01], external motivation, [*index* = 0.21 (95% CI, 0.07–0.43), *p* = 0.02], and (c) performance anxiety [*index* = 0.26 (95% CI, 0.08–0.56), *p* = 0.03], via CNF, was significant. These results support hypothesis 3, as the relation between PC and the outcomes was more evident as the moderator values increased, showed by conditional indirect effects that was significant at mean and high levels (+1 SD) of the moderator, but not at the low level (-1 SD).

**Table 2:** Modeling results of moderated mediation analyses testing hypothesis 2.

Direct effects	Mediator = competence need frustration			Dependent variable introjected^a^, external^b^, anxiety^c^		
		
	β	SE_β_	Two-tailed *p*-value	β	SEβ	Two-tailed *p*-value
Gender	–	–	–	0.01^a^	0.07^a^	0.85^a^
				0.03^b^	0.07^b^	0.66^b^
				0.16^c^	0.06^c^	0.02^c^
Domain	–0.13	0.06	0.03	0.11^a^	0.06^a^	0.10^a^
				0.29^b^	0.13^b^	0.02^b^
				–0.04^c^	0.06^c^	0.52^c^
Perfectionistic concerns	0.44	0.08	0.00	0.18^a^	0.08^a^	0.02^a^
				0.00^b^	0.08^b^	0.96^b^
				0.20^c^	0.09^c^	0.03^c^
Controlling conditions	0.26	0.09	0.00	0.11^a^	0.08^a^	0.16^a^
				0.23^b^	0.08^b^	0.00^b^
				0.04^c^	0.09^c^	0.65^c^
PC^∗^control	0.16	0.08	0.04	–0.07^a^	0.06^a^	0.21^a^
				–0.09^b^	0.07^b^	0.21^b^
				0.00^c^	0.07^c^	0.99^c^
Competence need frustration	–	–	–	0.41^a^	0.09^a^	0.00^a^
				0.42^b^	0.08^b^	0.00^b^
				0.31^c^	0.10^c^	0.00^c^

**Indirect effects**	**β**	**SEβ**	**Two-tailed *p*-value**	**CI 95% LL**		**CI 95% HL**

Conditional indirect effect of PC on introjected motivation^a^, external motivation^b^, anxiety^c^ through competence need frustration at:						
Low control (-1 SD)	0.18^a^	0.12^a^	0.14^a^	–0.02^a^		0.47^a^
	0.14^b^	0.10^b^	0.16^b^	–0.01^b^		0.37^b^
	0.16^c^	0.12^c^	0.18^c^	–0.02^c^		0.48^c^
Mean level of control	0.29^a^	0.11^a^	0.01^a^	0.10^a^		0.57^a^
	0.21^b^	0.09^b^	0.02^b^	0.06^b^		0.43^b^
	0.25^c^	0.12^c^	0.03^c^	0.08^c^		0.56^c^
High control (+1 SD)	0.39^a^	0.14^a^	0.00^a^	0.17^a^		0.72^a^
	0.29^b^	0.10^b^	0.00^b^	0.11^b^		0.50^b^
	0.35^c^	0.14^c^	0.01^c^	0.12^c^		0.69^c^


*All estimated parameters are standardized with STDYX Standardization, except the index of conditional effects that are only reported as unstandardized index (Hayes, 2017). ^*a*^Introjected; ^*b*^External; ^*c*^Anxiety.*

## Discussion

The purpose of this study was to examine why and under what circumstances perfectionistic concerns (PC) were associated with controlled motivation and performance anxiety in a sample of elite junior performers. We aimed to test the roles of controlling conditions and need frustration as explanatory mechanisms. In general, the results showed that the typical Norwegian elite junior performer experienced adaptive and well-functioning motivational processes. However, the results supported the vulnerability hypothesis of PC as a variable related to debilitative motivational processes. Furthermore, the current study tested and found support for the role of competence need frustration (CNF) as the key intervening variable between PC and the outcomes of (a) introjected motivation, (b) external motivation, and (c) performance anxiety. In addition, controlling teaching/coaching conditions were a moderator as the debilitative motivational processes tested in the three models were more evident among those reporting higher levels of controlling teaching/coaching conditions. Implications of these findings are discussed below and structured in line with the three hypotheses.

### The Debilitative Motivational Signature of PC Among Elite Junior Performers

The linking of the PC trait with SDT tenets both corroborated and extended previous perfectionism research. Supporting hypothesis 1, the results showed that higher levels of PC were positively associated with perceptions of controlling teaching/coaching style, the frustration of basic psychological needs, controlled motivation, and performance anxiety. This confirms initial evidence of PC as a contributor to SDT’s maladaptive motivational path, which is characterized by need frustration, controlled motivation, dysfunction, and ill-being (Boone et al., 2014; Hill, 2016; Jowett et al., 2016). These findings may indicate that the motivational signature of PC, particularly within TDEs, is the paradoxical portrayal of “successful failures,” characterized by conditional self-worth, self-critical attitudes, over-striving, and avoidance coping strategies (Eusanio et al., 2014; Hall, 2016; Patston and Osborne, 2016). That is, even elite junior performers, such as those sampled for this study, may end up feeling imperfect and as “failures” if they also possess high levels of PC, regardless of their quite extraordinary achievements (top 20% in their national age group). Linked with controlled motivation, and performance anxiety, such a motivational process certainly seems at odds with suggested guidelines for healthy TD (Hill A. et al., 2016).

### Need Frustration and the Role of Competence Need Frustration

The results partially supported hypothesis 2 and demonstrated that higher levels of PC were associated with introjected motivation and performance anxiety both directly and, more strongly, indirectly through CNF. External motivation had only indirect associations. These findings fit nicely alongside recent work in sport psychology that has clarified basic needs as intervening variables in the relation between perfectionism and burnout (Mallinson and Hill, 2011; Jowett et al., 2016). Our findings also extend these studies by testing other outcomes known to undermine optimal functioning and well-being in elite junior performers, such as controlled motivation and performance anxiety (Woodman and Hardy, 2003; Kenny et al., 2004; De Meyer et al., 2016; Correia and Rosado, 2018).

The results add interesting nuances to previous studies of needs frustration (Mallinson and Hill, 2011; Boone et al., 2014; Jowett et al., 2016), as only the need for competence functioned as an intervening variable between PC on the one hand, and controlled motivation and performance anxiety on the other. There were also positive associations between PC and frustration of the other two needs (i.e., autonomy and relatedness). However, no significant interaction effect, or indirect associations on the outcomes, were found. As such, the need for competence turned out to be the key psychological need in the current sample of elite junior performers. As found in other TDEs studies (e.g., Fransen et al., 2018a,b; Stabell, 2018) competence seems to be the most important “currency” in TDEs. As the very essence of TDEs is to demonstrate superiority and outperform others, further possibilities (i.e., social status, attention, re-selection, and advantages) are seemingly dependent on achieved success (Stabell, 2018). Hence, elite junior performers reporting higher levels of PC are likely to get their inherited vulnerability and conditional self-worth activated when operating within TDEs (Hall, 2016). To avoid inferiority and failure, elite junior performers reporting higher levels of PC might end up in a debilitative motivational circle of emotions (i.e., frustration, stress, and negative affect), cognition (i.e., guilt, shame, and fear of failure), and behavior (i.e., rigidity, obsession, and avoidance strategies), constantly nurturing their CNF, controlled motivation, and performance anxiety (Flett and Hewitt, 2016). Due to the cross-sectional nature of the study, this is initial evidence, and we suggest the need for future studies extending this line of perfectionism research with longitudinal designs.

Concerning explanations of why CNF and not autonomy and relatedness did intervene between PC and the outcomes of controlled motivation and performance anxiety in the current study, one might only speculate. For example, one explanation might be the unique and vital role competence holds, not only as a core driver of PC (Hill, 2016), but also within the three tested outcomes. Performance anxiety is triggered by an experienced imbalance between situational demands and perceived competence (Correia and Rosado, 2018). Also in SDT, the origin of positive functioning and autonomous motivation (opposite to controlled motivation) are tied to competence, to the innate urge to interact effectively and master one’s surroundings (Elliot et al., 2002). Moreover, competence is especially activated in TDEs, where competence seems to be the currency that controls the conditional regard inherited in the controlling teaching/coaching style (Stabell, 2018; Haraldsen et al., in press), resulting in a strong conceptual coherence between the study variables in the model where CNF are used as the explanatory mechanism. More research is needed to extend this line of SDT-based research in diverse contexts.

### The Moderating Role of Controlling Conditions

The interaction between PC and controlling teaching/coaching conditions has been less studied compared to PC and parenting styles (Soenens et al., 2012; Assor et al., 2014). Hence, the current study tested whether tendencies typically associated with parenting style (an origin of PC), could be extended to the teaching/coaching setting in TDEs. The results indicated that this was the case, as the interaction between PC and controlling teaching/coaching conditions (Bartholomew et al., 2018), were associated with higher levels of CNF, controlled motivation, and performance anxiety.

When reporting high levels of PC, elite junior performers might be biased in the way they perceive their teaching/coaching styles (Appleton et al., 2011; Boone et al., 2014; Nordin-Bates et al., 2014). Activated by aspects of controlling conditions, they are likely to enter a kind of hypervigilant state, driven by emotional stress from their conditional self-worth, which in turn, seems to associate with fear of failure and avoidance motivation (Shafran et al., 2002). Controlling conditions might reinforce this pattern, as a trigger and extension of conditional regard received from another significant other (Assor et al., 2014). The displaying of higher levels of PC might also function as a substitute for being externally controlled, as a way of taking the control back, directing it into self-control, obsessiveness, and relentless pursuit for success (Shafran et al., 2002; Boone et al., 2014). Thus, such behavior might trigger and increase the PC tendencies within performers, whereas, when faced with low controlling conditions these tendencies might be immobilized (Shafran et al., 2002; Nordin-Bates et al., 2014).

From an applied perspective, the most vital lesson learned from this study might be the importance of avoiding controlling mechanisms. This seems especially true in ambitious performance-oriented TDE settings, where too many performers are likely to experience higher levels of PC, as well as risking failure and adversity (Dunn et al., 2012; Appleton and Curran, 2016; Schinke et al., 2017). Moreover, teachers/coaches should be encouraged to pay attention to how they as authority figures and gatekeepers (Nash and Collins, 2006; Burwell, 2013), indirectly (and perhaps unintentionally) hold power, and thus might pressure, control, and affect elite junior performers’ motivation in conditional and, hence, debilitative directions. As an alternative, and in line with the SDT tenets, they should be stimulated and taught how to behave in less controlling and in more autonomy-supportive ways, as research indicates that autonomous functioning might be a proactive coping strategy and resilience factor (Radel et al., 2013; Ryan and Deci, 2017; Ryan and Ryan, 2018).

### Strengths, Limitations, and Future Research

The findings should be interpreted in light of some limitations. The cross-sectional design, preventing temporal precedence, hampers absolute evidence of the order of variables or the stability of the indirect associations tested. Another limitation originates from the sole reliance on self-report data, which may be a threat to validity. The sample size (*N* = 171) might be a limitation from a statistical perspective; however, the sample is also a strength, as it accounts for almost all of the unique and exclusive top 20% high-achieving population of elite junior performers in Norway (response rate = 84%). Strengths are also the novel and sophisticated conditional process modeling (for details, see Hayes, 2017), linking controlling teaching/coaching style with perfectionism, and hence, providing deeper insight into the motivational signature of perfectionism in elite junior performers. Thus, future studies should re-examine similar models longitudinally with larger samples from different domains and TDE settings.

## Conclusion

Framed within SDT, the present study examined the motivational signature of PC in a sample of Norwegian elite junior performers from sport and arts. The results indicated that displaying high levels of PC might expose elite junior performers to higher risks of experiencing debilitative motivational processes. Specifically, they appear more likely to develop controlled motivation and experience performance anxiety through competence need frustration (CNF). Furthermore, the findings indicated that these experiences were conditional on varying levels of reported controlling teaching/coaching conditions. Hence, the indirect associations on controlled motivation and performance anxiety via CNF was more evident in performers reporting mean and higher levels of controlling teaching/coaching conditions. In contrast, there were no indirect associations via CNF for those performers who reported low levels of controlling conditions. Overall, these findings support key tenets of SDT and implies that coaches/teachers of elite junior performers might play a key role in preventing CNF and experiences of debilitative motivational processes through avoiding the misuse of a controlling teaching/coaching style.

## Data Availability

The datasets generated for this study are available on request to the corresponding author.

## Ethics Statement

This study was carried out in accordance with the recommendations of Norwegian Center for Research Data. All subjects gave written informed consent in accordance with the Declaration of Helsinki. The protocol was approved by Marianne Høgetveit Myhren and Eva J. B. Payne. Participants voluntarily consented to participate after receiving oral and/or written information about the study. All data are anonymous and ethical procedures for storage of data are followed.

## Author Contributions

HMH has been the main contributing researcher in this study. HMH, HH, BS, FA, and SN-B conceived and designed the study, drafted the work and revised the content, and approved the final version of the manuscript and agreed the research quality. HMH, HH, and BS analyzed the data.

## Conflict of Interest Statement

The authors declare that the research was conducted in the absence of any commercial or financial relationships that could be construed as a potential conflict of interest.
