# Supplementary material for: The Role of Perfectionism and Controlling Conditions in Norwegian Elite Junior Performers’ Motivational Processes
Source: Front Psychol. 2019 Jun 12;10:1366. doi: 10.3389/fpsyg.2019.01366 (PMC6582430; doi:10.3389/fpsyg.2019.01366)
Supplement: Supplementary file 1 [file Table_1.DOCX]

**Supplemental material to “The Role of Perfectionism and Controlling Conditions in Norwegian Elite Junior performersʼ Motivational Processes”**

**Validation of Measurement scales**

An overall confirmatory factor analysis (CFA) of all the study variables showed acceptable fit (χ^2^ (565) = 860.13, *p* = .00, χ^2^/df = 1.5, CFI = .90, SRMR = .06, RMSEA = .06 [90% CI, .048- .062]) after the adjustments in the validation process of each sub-scale outlined below. The subscale Concern over mistakes showed poor model fit and we had to make a reduced and adjusted subscale. Other studies using the Frost Multidimensional Perfectionism Scale (F-MPS; Frost, Marten, Lahart, & Rosenblate, 1990) have faced similar challenges with cross-loadings and/or low loadings on their respective subscales, and have successfully used reduced and adjusted sub-scales (Cox, Enns, & Clara, 2002). The remaining study variables faced only minor adjustments.

**Concern over Mistakes**

The 9-item subscale Concern over mistakes (CM) from the F-MPS (Frost et al., 1990) did not provide an acceptable fit to the data in the initial CFA (CFI = .84, SRMR = .07, RMSEA = .13 [90% CI, .10- .15]). In line with outlined suggestions (Cox et al., 2000), we inductively developed a reduced and adjusted subscale. To guide this approach, we combined exploratory factor analysis (EFA) and theoretical interpretation (Tabachnick & Fidell, 2007). Because our aim was to test if the scale consisted of one or two separate factors, a Varimax rotation was chosen to best serve our purpose (Hair, Anderson, Tatham, & William, 1998; Tabachnick & Fidell, 2007).

The EFA with Varimax rotation suggested a two-factor solution (51.40% total variance explained) supported by Kaiserʼs criteria (Eigenvalues > 1) and the scree plot (Tabachnick & Fidell, 2007). The Kaiser-Meyer-Olkin (KMO) test on the rotated solution was excellent (KMO = .876, Bartlettʼs test of Spehricity; *p* ˂ .01), indicating a highly valid EFA (Hair et al., 1998; Tabachnick & Fidell, 2007). The rotated factor matrix and the item wordings are displayed in Table A1, and showed that the scale divided into two separate dimensions of CM instead of the original one dimension. The first factor reflected perfectionism driven by conditional self-worth (items CM1-CM4), and the second factor mirrored internal distress over making mistakes and not being the best (items CM6-CM9). CM7 and CM8 loaded on both factors; however, loadings were just above .3 in factor one, and above .7 on factor two, indicating a better fit within factor two. One item (CM5; “If I fail at my activity, I feel like I am a failure as a person”) loaded highly (above .4) on both factors. However, when interpreting the content of this item, it is conceptually related to conditional self-worth. When examining the factor loadings on the CM self-worth sub-scale (see Table A2), including CM5, the factor structure was good and supported our placing of CM5 in the CM self-worth factor. The CFA of CM self-worth (5 items; CM1-CM5) supported this decision further, as it showed excellent fit (CFI = .99, SRMR = .02, RMSEA = .02 [90% CI, .00- .11]). The CFA of CM internal distress (4 items; CM6-CM9; CFI = .99, SRMR = .02, RMSEA = .06 [90% CI, .00- .17]) did also perform well.

These two dimensions share similar characteristics with Hewitt, Flett, Turnbull-Donovan, and Mikailʼs (1991) two subscales of self-oriented (CM internal distress) and socially prescribed perfectionism (CM self-worth), and are a theoretically sound split between perfectionistic concerns that are mainly internal and personal versus external and socially driven (Appleton & Curran, 2016; Hewitt et al., 1991; Madigan & Stoeber, 2016). Moreover, socially prescribed perfectionism is nurtured by conditional regard and contingent self-worth (Appleton & Curran, 2016; Hewitt et al., 1991) which conceptually aligns with items CM1-CM5.

**Doubts about Actions**

The 4 item sub-scale of Doubts about actions (DA) showed good fit in the CFA (CFI = .99, SRMR = .03, RMSEA = .06 [90% CI, .00- .18]).

**The Predictor Variable of Perfectionistic Concerns Composite Scale**

Over the past 25 years, empirical evidence suggests that Perfectionistic Concerns (PC) is a higher order dimension of perfectionism that conceptually comprises combinations of several lower-order perfectionism facets (i.e., concern over mistakes, doubts about actions and fear of negative social evaluation) measured by several instruments (e.g., F-MPS, HF-MPS, S-MPS, and MIPS; Madigan & Stoeber, 2016; Smith, Saklofske, Stoeber, & Sherry, 2016). Thus, as PC are often comprised of more than one latent factors, we intended the most comprehensive, but still valid, representation of PC (Hill, 2016). In order to get a broader conceptualization of PC than half the CM scale, we tried to use a combination of the CM and DA subscales. Combining items from CM and DA in a merged scale have been used in previous studies in sport, dance, and exercise contexts (Cox et al., 2002; Madigan & Stoeber, 2016). Guided by theoretical, empirical, and comparability reasons, we ended up applying a composite score of the CM self-worth scale and the full DA scale. The choice to use CM self-worth (vs CM internal distress) was based on theoretical arguments. Specifically, the CM self-worth sub-dimension is theoretically more aligned with overall PC and controlling conditions, which both are conceptually underpinned by conditional self-worth (Assor, Kanat-Maymon, & Roth, 2014; DiBartolo, Frost, Chang, LaSota, & Grills, 2004; Soenens & Vansteenkiste, 2010). Moreover, CM self-worth is a reflection of socially described perfectionism (Hill, 2016), also a sub-dimension of PC within Hewitt & Flettʼs perfectionism instrument (Hewitt et al., 1991). The CFA of the composite PC scale showed an acceptable fit (CFI = .95, SRMR = .06, RMSEA = .07 [90% CI, .03-.11]), and hence, this scale was used in the tested models of moderated mediation.

**The Moderator Controlling Conditions**

In the moderator variable controlling conditions we had to remove one of the items of the scale that caused problems as it turned out to be a so-called Heywood case. The item, when investigated in EFA, produced an additional factor, resulting in one autonomy frustration factor and one factor representing the merger of competence- and relatedness frustration. When forced into a one-factor solution, this item's communality exceeded 1.0. After removing this item the CFA performed very well (CFI = 1.00, SRMR = .03, RMSEA = .00 [90% CI, .00-.09]).

**The Intervening Variable Association of Basic Need Frustration**

The scale showed good fit for both autonomy need frustration (CFI = .99, SRMR = .02, RMSEA = .07 [90% CI, .00- .18]) and relatedness need frustration (CFI = .99, SRMR = .02, RMSEA = .07 [90% CI, .00- .18]). In the competence need frustration scale, however, there were high cross-loadings between two items, and the CFA model fit was poor (CFI = .88, SRMR = .05, RMSEA = .25 [90% CI, .16- .34]). However, after we removed one item with high cross-loadings, the CFA of competence need frustration had only three indicators (just- identified) and goodness-of-fit evaluation did not apply (Brown, 2014). However, the overall CFA of the three basic need frustration sub-scales showed an acceptable fit (CFI = .96, SRMR = .05, RMSEA = .06 [90% CI, .02- .08]).

**The Outcomes of Performance Anxiety and Controlled Motivation**

The outcome variables were measured in line with the original instruments without any adjustment. The CFA of introjected motivation (CFI = .96, SRMR = .05, RMSEA = .06 [90% CI, .02- .08]) and external motivation (CFI = .96, SRMR = .05, RMSEA = .06 [90% CI, .02- .08]) showed good fit. However, as performance anxiety had only three indicators, it resulted in a just-identified model (*df* = 0), and goodness-of-fit evaluation did not apply (Brown, 2014). Factor loadings ranged from .59-.97, which is regarded as acceptable in the statistical literature (Brown, 2014).

**References**

Appleton, P. R., & Curran, T. (2016). The origins of perfectionism in sport, dance, and exercise. In A.P. Hill. (ed.), *The Psychology of Perfectionism in Sport, Dance and Exercise* (pp. 57-81). New York: Routledge.

Assor, A., Kanat-Maymon, Y., & Roth, G. (2014). Parental conditional regard: Psychological costs and antecedents. In N. Weinstein (ed.), *Human Motivation and Interpersonal Relationships* (pp. 215-237). Dordrecht: Springer.

Brown, T. A. (2014). *Confirmatory factor analysis for applied research.* New York: Guilford Publications.

Cox, B. J., Enns, M. W., & Clara, I. P. (2002). The multidimensional structure of perfectionism in clinically distressed and college student samples. *Psychological Assessment, 14*, 365-373. doi.org/10.1037/1040-3590.14.3.365

DiBartolo, P. M., Frost, R. O., Chang, P., LaSota, M., & Grills, A. E. (2004). Shedding light on the relationship between personal standards and psychopathology: The case for contingent self-worth. *Journal of Rational-Emotive and Cognitive-Behavior Therapy, 22*, 237-250. doi.org/10.1023/B:JORE.0000047310.94044.ac

Frost, R. O., Marten, P., Lahart, C., & Rosenblate, R. (1990). The dimensions of perfectionism. *Cognitive Therapy and Research, 14*, 449-468.

Hair, J. F., Anderson, R. E., Tatham, R. L., & William, C. (1998). Multivariate data analysis. Upper Saddle River, NJ: Prentice Hall.

Hewitt, P. L., Flett, G. L., Turnbull-Donovan, W., & Mikail, S. F. (1991). The Multidimensional Perfectionism Scale: Reliability, validity, and psychometric properties in psychiatric samples. *Psychological Assessment: A Journal of Consulting and Clinical Psychology, 3*, 464. doi:10.1037/1040-3590.3.3.464

Hill, A. P. (2016). Conceptualizing perfectionism. In A.P. Hill. (ed.), *The Psychology of Perfectionism in Sport, Dance and Exercise* (pp. 3-30). New York: Routledge.

Madigan, D. J., & Stoeber, J. (2016). Measuring perfectionism in sport, dance, and exercise: Review, critique, recommendations *The Psychology of Perfectionism in Sport, Dance and Exercise* (pp. 47-72): Routledge.

Smith, M. M., Saklofske, D. H., Stoeber, J., & Sherry, S. B. (2016). The big three perfectionism scale: A new measure of perfectionism. *Journal of Psychoeducational Assessment, 34*, 670-687. doi.org/10.1177/0734282916651539

Soenens, B., & Vansteenkiste, M. (2010). A theoretical upgrade of the concept of parental psychological control: Proposing new insights on the basis of self-determination theory. *Developmental Review, 30*, 74-99. doi:10.1016/j.dr.2009.11.001

Tabachnick, B. G., & Fidell, L. S. (2007). *Using multivariate statistics.* Harlow: Pearson.

Table A1

*Rotated Factor Matrix of Concern over mistakes subscale 9 items*

|  | Item (back-translated from Norwegian to English) | Factor 1 | Factor 2 |
| --- | --- | --- | --- |
| CM1 | Coaches/teachers/peers will probably think less of me if I make a mistake. | .745 |  |
| CM2 | If I do not do as well as other in my activity, it means I am an inferior being. | .651 |  |
| CM3 | If I do not do well all the time in my activity, people will not respect me. | .770 |  |
| CM4 | The fewer mistakes I make in my activity, the more people will like me. | .573 |  |
| CM5 | If I fail at my activity, I feel like I am a failure as a person. | .439 | .503 |
| CM6 | I am usually upset if I make a mistake when I practice my activity. |  | .628 |
| CM7 | If someone does a task at my activity better than I do, then I feel as if I failed the whole task. | .336 | .730 |
| CM8 | If I fail partly fail in my activity, it is as bad as being a complete failure. | .313 | .702 |
| CM9 | I hate being less than the best at things in my activity. |  | .596 |

*Note.* Extraction Method: Principal Axis Factoring. Rotation Method: Vairmax with Kaiser Normalization. Coefficients below .03 are suppressed.

Table A2:

*Factor Loadings of Concern over mistakes self-worth 5 items*

|  | Item (back-translated from Norwegian to English) | Factor 1 |
| --- | --- | --- |
| CM1 | Coaches/teachers/peers will probably think less of me if I make a mistake. | .794 |
| CM2 | If I do not do as well as other in my activity, it means I am an inferior being. | .672 |
| CM3 | If I do not do well all the time in my activity, people will not respect me. | .780 |
| CM4 | The fewer mistakes I make in my activity, the more people will like me. | .649 |
| CM5 | If I fail at my activity, I feel like I am a failure as a person. | .586 |

*Note.* Derived from the CFA in Mplus.
